# Frequency and typing of *Propionibacterium acnes* in prostate tissue obtained from men with and without prostate cancer

**DOI:** 10.1186/s13027-016-0074-9

**Published:** 2016-06-09

**Authors:** Sabina Davidsson, Paula Mölling, Jennifer R. Rider, Magnus Unemo, Mats G. Karlsson, Jessica Carlsson, Swen-Olof Andersson, Fredrik Elgh, Bo Söderquis, Ove Andrén

**Affiliations:** Department of Urology, Faculty of Medicine and Health, Örebro University, Örebro, Sweden; Department of Laboratory Medicine, Clinical Microbiology, Faculty of Medicine and Health, Örebro University, Örebro, Sweden; Department of Epidemiology, Harvard T.H. Chan School of Public Health, Boston, MA USA; Channing Division of Network Medicine, Department of Medicine, Brigham and Women’s Hospital and Harvard Medical School, Boston, MA USA; Department of Laboratory Medicine, Pathology, Örebro University Hospital, Örebro, Sweden; Department of Clinical Microbiology, Umeå University, Umeå, Sweden; Faculty of Medicine and Health, Örebro University, Örebro, Sweden; A Member of the Transdisciplinary Prostate Cancer Partnership (TopCaP), Örebro, Sweden; Department of Urology, Örebro University Hospital, SE-701 85 Örebro, Sweden

**Keywords:** Prostate cancer, *Propionibacterium acnes*, Infection, Inflammation

## Abstract

**Background:**

Prostate cancer is the most common cancer among men in Western countries but the exact pathogenic mechanism of the disease is still largely unknown. An infectious etiology and infection-induced inflammation has been suggested to play a role in prostate carcinogenesis and *Propionibacterium acnes* has been reported as the most prevalent microorganism in prostatic tissue. We investigated the frequency and types of *P. acnes* isolated from prostate tissue samples from men with prostate cancer and from control patients without the disease.

**Methods:**

We included 100 cases and 50 controls in this study. Cases were men diagnosed with prostate cancer undergoing radical prostatectomy and controls were men undergoing surgery for bladder cancer without any histological findings of prostate cancer. Six biopsies taken from each patient’s prostate gland at the time of surgery were used for cultivation and further characterization of *P. acnes*.

**Results:**

The results revealed that *P. acnes* was more common in men with prostate carcinoma than in controls, with the bacteria cultured in 60 % of the cases vs. 26 % of the controls (*p* = 0.001). In multivariable analyses, men with *P. acnes* had a 4-fold increase in odds of a prostate cancer diagnosis after adjustment for age, calendar year of surgery and smoking status (OR: 4.46; 95 % CI: 1.93–11.26). To further support the biologic plausibility for a *P. acnes* infection as a contributing factor in prostate cancer development, we subsequently conducted cell-based experiments. *P. acnes-* isolates were co-cultured with the prostate cell line PNT1A. An increased cell proliferation and cytokine/chemokine secretion in infected cells was observed.

**Conclusion:**

The present study provides further evidence for a role of *P. acnes* in prostate cancer development.

**Electronic supplementary material:**

The online version of this article (doi:10.1186/s13027-016-0074-9) contains supplementary material, which is available to authorized users.

## Background

Prostate cancer (PCa) is the most common malignant neoplasm among men in Western industrialized countries and the second most common cancer diagnosed among men worldwide [1, 2]. The exact pathogenic mechanism of the disease is still largely unknown. However, chronic inflammation has been hypothesized to affect prostate carcinogenesis [3]. Both chronic and acute inflammation are commonly observed upon examination of histological samples [4]. This inflammation is present throughout the entire gland, not only in close proximity to the tumor, and thus is unlikely to be explained entirely as an immune response to malignancy [5]. The multiple foci of inflammation observed are suggestive of an infectious etiology and infection-induced inflammation has been hypothesized to play a role in prostate carcinogenesis [6].

*Propionibacterium acnes* is a Gram-positive bacillus that forms part of the normal flora of the skin. Although generally regarded as exhibiting low pathogenic potential, *P. acnes* has been recognized as contributing to the pathogenesis of acne vulgaris [7]. An increasing number of reports have implicated the microorganism as an opportunistic pathogen responsible for a wide range of low-grade infections such as orthopaedic implant device related infections [8], prosthetic valve endocarditis [9], sternal wound infections after cardiothoracic surgery [10, 11], and shunt-associated central nervous system infections [12]. Several independent studies have recently reported a high frequency of *P. acnes* in prostate tissue samples, both from men with prostatitis and PCa [13–17]. Moreover, isolation of *P. acnes* has been positively associated with the presence and extent of both acute and chronic inflammation [14]. In addition, previous studies have shown that *P. acnes* has the capability to stimulate prostate epithelial cell lines to secret cytokines and chemokines, such as Interleukin 6 (IL-6) and Chemokine (C-X-C motif) ligand 8 (IL-8) [15, 18, 19]. These inflammatory markers are critical for a sustained inflammatory response and both are thought to play an important role in the development of different types of cancers, among them PCa [20, 21]. Furthermore, two different in vivo models have recently been developed supporting the hypothesis of *P. acnes* as a contributing agent for prostatic inflammation [22, 23].

Further evidence supporting the hypothesis of *P. acnes* as a contributing factor in PCa development was published by Fassi Fehri et al., showing transformation of prostate cells after being infected with *P. acnes* [15], and from epidemiologic studies showing that men treated with tetracycline, commonly indicated for acne, were more likely to be subsequently diagnosed with prostate cancer [24].

The contradiction between the role as a skin commensal and the more pathogenic behavior might be partly explained by strain-specific properties where different *P. acnes* strains contribute differently to health and disease.

*P. acnes* strains can be divided into the major types IA, IB, II, and III according to sequence comparison of the *recA* or *tly* genes [25]. More recently, further discrimination has been aided by various multilocus sequence typing (MLST) schemes and repetitive-sequence-based PCR protocols [26–29]. *P. acnes* subtype I, more specifically termed I-1a, is predominantly associated with moderate to severe acne [27, 28]. In contrast *P. acnes* type II is reported as the most prevalent type in previous studies of prostatic specimens obtained from patients with PCa [14]. However, a major limitation in studies performed to investigate the association between *P. acnes* and PCa has been the lack of appropriate control tissue i.e. samples from men without prostate malignancy.

In the present study we investigate the presence and types of *P. acnes* strains isolated from multiple peri-operative prostate tissue samples from men with PCa and from control patients with no histological evidence of the disease. *P. acnes* was more in men with prostate cancer then in controls, with the bacteria cultured in 60 % of the cases vs. 26 % of the controls. To explore how *P. acnes* might contribute to an increased risk for PCa initiation, we further investigated the capacity of *P. acnes* to modulate proliferation and secretion of inflammatory mediators in prostate cells. The results showed an increased proliferation and cytokine/chemokine secretion in prostate cells infected with *P. acnes*.

## Results

### Isolation of *P. acnes* from prostate tissue samples

Multiple prostate tissue samples were collected from 100 cases and 87 controls. However, 37 patients those initially was categorized as controls displayed signs of PCa at the pathological anatomical examination and were subsequently considered as a separate group (controls with PCa). Clinical characteristics for all cases and controls without PCa are shown in Table 1.

**Table 1:** Selected characteristics of men with and without prostate cancer

	Cases	Controls	*P*-value	Controls with PCa^a^
	*N* = 100	*N* = 50		*N* = 37
Age at surgery (yrs), Mean (STD)	64.0 (4.4)	65.7 (7.1)	<0.0001	70.2 (2.1)
Year of surgery, Median (Min-Max)	2010 (2008–2011)	2011 (2009–2015)	0.002	2013 (2009–2015)
History of smoking, N (%)	8 (8.3)	7 (14.6)	0.24	
*P. acnes* present, N (%)	60 (60.0)	13 (26.0)	<0.0001	13 (35.1)
Surgical Gleason Score, N (%)		--	--	
2-5	0 (0.0)			4 (5.8)
6	34 (34.0)			28 (75.7)
7	60 (60.0)			4 (10.8)
8-9	6 (6.0)			1 (2.7)
pTNM stage, N (%)		--	--	
2	85 (85.9)			--
3	14 (14.1)			--

^a^Compared to cases, Chi-square *p*-value for P. acnes present = 0.01 and Mantel-Haenszel Chi-Square *p*-value for surgical Gleason score < 0.0001

The predominant microorganism obtained from the prostatic tissue was *P. acnes*. Less frequent isolates included coagulase negative staphylococcus and only single isolates of other species such as *Corynebacterium species* and *Streptococcus species*, similar to previous studies [16, 29]. From the entire cohort, 182 bacterial isolates were characterized as *P. acnes*. The bacteria were identified in 60 % of the cases (60/100), a significantly greater proportion compared to controls (13/50; 26 %) (*p* = 0.001). In controls with PCa, *P. acnes* was found in 13/37 (35 %). In cases, there was no association between *P. acnes* status and tumor stage (*p* = 0.43). Median PSA levels at diagnosis were similar in P. acnes-positive and P. acnes-negative cases (6.75 vs. 6.0 ng/mL, respectively; *p* = 0.54). Cases had Gleason scores reflecting more poorly differentiated tumors compared to controls incidentally found to have tumor (Table 1; *p* < 0.0001). Among all cases and controls with incidental tumor, there was no association between Gleason score and *P. acnes* status.

As shown in Table 2, in a univariate logistic regression model, men with *P. acnes* had a statistically significant more than 4-fold increase in odds of PCa compared to men without the bacterium. The association remained statistically significant and became more pronounced after adjustment for age at surgery, year of surgery and smoking status (OR: 4.46; 95 % CI: 1.93–11.26).

**Table 2:** Odds ratios (95 % confidence intervals) for prostate cancer according to *P. acnes* in prostate tissue

	Crude Model	Model 1	Model 2^a^
*P. acnes*	4.27 (2.02–9.02)	3.66 (1.61–8.30)	4.67 (1.93–11.26)
Age at surgery, yrs (continuous)	--	0.95 (0.88–1.02)	0.91 (0.84–0.99)
Year of surgery (continuous)	--	0.48 (0.33–0.68)	0.46 (0.31–0.67)
Smoker	--	--	0.59 (0.15–2.34)

^a^5 subjects missing data on smoking excluded from analysis

**Fig. 1:**
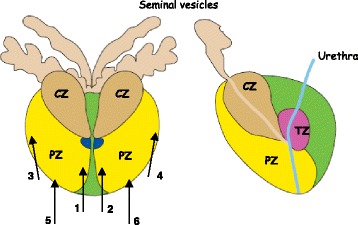
Zonal anatomy of the prostate. Zonal anatomy of the human prostate, divided into three glandular zones: central zone (CZ), peripheral zone (PZ) and transition zone (TZ). The arrows indicate were the 6 biopsies are taken

To investigate the distribution of *P. acnes* in prostate tissue, 6 biopsies were taken from each patient according to a specific scheme (Fig. 1). In men with PCa, *P. acnes* was identified in 134 biopsies, representing a median of 2 isolates per person (134/60). In controls *P. acnes* was identified in 19 biopsies, equivalent to a median number of 1 isolate per person (19/13). The corresponding numbers for the controls with PCa were 29 positive biopsies representing a median of 2 isolates per person (29/13). Cases had a significantly higher number of *P. acnes* positive biopsies compared to the controls (*p* = 0.01). Furthermore, a significant difference in the number of positive biopsies was also found between the controls and the controls with PCa (*p* = 0.04).

We also investigated the distribution of *P. acnes* in different prostate zones were the locations within zones were estimated based on gross examination. Biopsy 1 and 2 represented the transition zone, 3 and 4 the peripheral zone, and 5 and 6 the apex area (from Fig. 1). In cases isolates appeared to be more evenly distributed anatomically compared to the controls where infection was more likely to be present in the transition zone, even though not statistically significant (*p* = 0.07). The results are presented in Table 3.

**Table 3:** Presence and distribution of *P. acnes* in cases, controls, and controls with prostate cancer (PCa)

	Cases	Controls	Controls with PCa
	*N* = 100	*N* = 50	*N* = 37
*P. acnes* present	60 (60 %)	13 (26 %)	13(35 %)
*P. acnes* present in			
Biopsy I	27 (20 %)	4 (21 %)	3 (10 %)
Biopsy II	21 (16 %)	4 (21 %)	4 (14 %)
Biopsy III	20 (15 %)	3 (16 %)	6 (21 %)
Biopsy IV	24 (18 %)	4 (21 %)	6 (21 %)
Biopsy V	25 (18 %)	3 (16 %)	5 (17 %)
Biopsy VI	17 (13 %)	1 (5 %)	5 (17 %)

### Typing based on *tly*

The different subtypes of the *P. acnes* isolates were investigated by amplifying and sequencing the putative hemolysin gene *tly*, which was successfully performed in all 182 isolates. *P. acnes* type I and II were the most common lineages, detected at a similar ratio in both cases and controls (Additional file 1). In controls with prostate cancer, *P. acnes* type I was the most frequent lineage. We further investigated whether a single prostate gland could be infected with multiple *P. acnes tly* types. Among patients with more than one biopsy positive for *P. acnes,* two different types were present in approximately 50 % (21/41).

### Typing based on DiversiLab

The 182 prostatic *P. acnes* isolates could successfully be typed by using the repetitive-sequence-based PCR method (DiversiLab). Based on a minimum of 95 % similarity and with a difference of up to one band in the dendrogram, typing separated the prostatic isolates into the three major *tly* types (I, II, and III) with only a few exceptions. The method differentiated the isolates into a total number of 23 rep-PCR fingerprint patterns (Fpp) shown in Additional file 1, where the most common Fpp:s were 1, 2, 3, 6, 9, and 11 containing 13, 53, 12, 22, 17, and 11 *P. acnes* isolates respectively.

### Wound-healing and proliferation assays

Wound-healing assays were performed in order to investigate if an infection with *P. acnes* type IA or II could have an effect on the migration or proliferation of prostate epithelial cells. The results indicated that cells infected with *P. acnes* type IA or II closed the wound faster compared to uninfected cells. If the infected cells were treated with Mitomycin C, this effect was not seen, which indicates that a *P. acnes* infection in prostate epithelial cells have an effect on proliferation, not migration. In order to quantitate potential differences in proliferation between infected and uninfected cells, as well as between different MOI of *P. acnes,* a proliferation assay was used. PNT1A had an increased proliferation when infected with *P. acnes* type IA or II for 24–48 h (Table 4). However, none of the differences in proliferation were statistically significant.

**Table 4:** The percentage difference in proliferation seen in PNT1A cells infected with *P. acnes* type IA or II for 24 – 48 h, compared to uninfected cells

Time of infection	MOI	Type IA difference (%)	Type II difference (%)
24 h	5	+25.1	+45.0
	10	+42.9	+9.3
	20	+44.8	+19.8
	50	+39.0	−6.2
48 h	5	+33.1	+14.5
	10	+24.8	+37.2
	20	+15.7	+48.4
	50	+29.6	+44.5

### Short-term infections

**Fig. 2:**
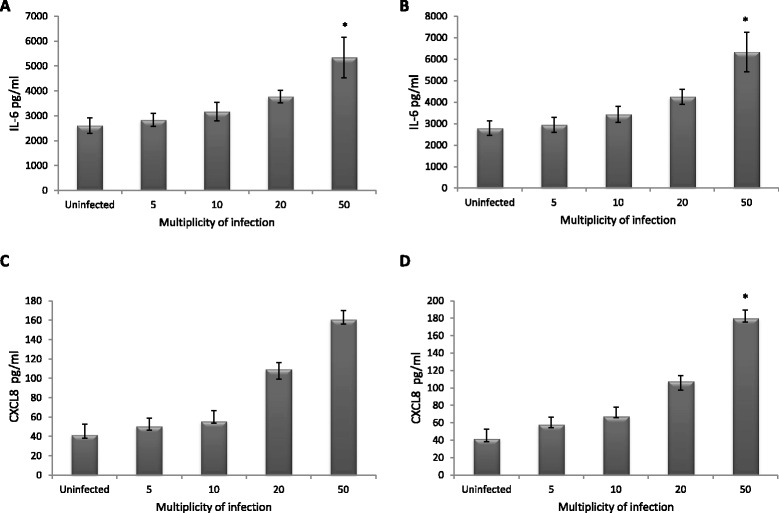
Secretion of IL-6 and CXCL8 from PNT1A cells infected with *P. acnes*. Secretion of IL-6 and CXCL8 from PNT1A cells infected with *P. acnes* type IA or II at different MOI. **a** IL-6 secretion from cells infected with *P. acnes* type I for 48 h. **b** IL-6 secretion from cells infected with *P. acnes* type II for 48 h. **c** CXCL8 secretion from cells infected with *P. acnes* type I for 48 h. **d** CXCL8 secretion from cells infected with *P. acnes* type II for 48 h. * Statistical significant difference in secretion compared to uninfected cells (*p <* 0.05 after adjustments for multiple testing)

In order to investigate if an infection with *P. acnes* type IA or II could modulate the secretion of pro-inflammatory mediators from prostate cells, the concentrations of IL6 and CXCL8 were measured in cell-media from cells infected with *P. acnes* and compared to uninfected cells. When infecting PNT1A cells with *P. acnes* type IA or II at different MOI for 48 h, a dose response effect could be seen for the secretion of both IL-6 and CXCL8, with an increasing secretion with increasing MOI (Fig. 2). There were a statistically significant difference in the secretion of IL-6 between uninfected cells and cells infected with 50 MOI of *P. acnes* type IA or II (*p =* 0.035, Fig. 2a and b). There were also a statistically significant difference in the secretion of CXCL8 between uninfected cells and cells infected with 50 MOI of *P. acnes* type II (*p* = 0.01, Fig. 2d).

### Long-term infections

**Fig. 3:**
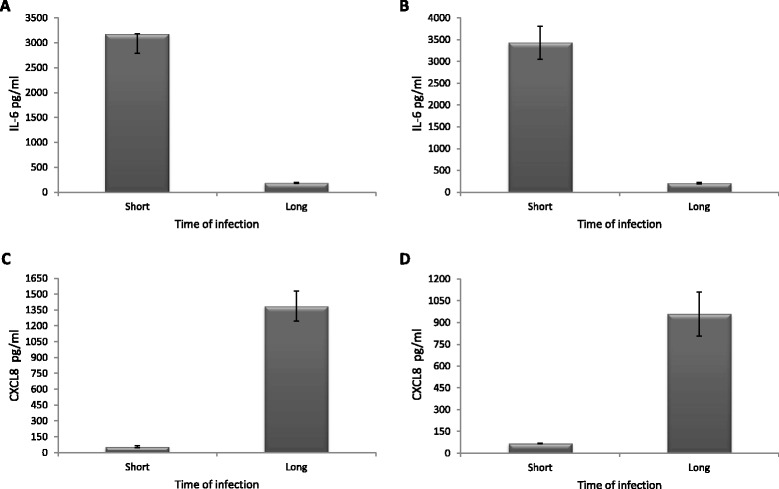
Secretion of IL-6 and CXC8 secretion between short and long-term infection. Differences in IL-6 and CXCL8 secretion between short- (48 h) and long-term (1 week) infections of PNT1A cells infected with *P. acnes* type IA or II. **a** IL-6 secretion from cells infected with *P. acnes* type I for 48 h or 1 week. **b** IL-6 secretion from cells infected with *P. acnes* type II for 48 h or 1 week. **c** CXCL8 secretion from cells infected with *P. acnes* type I for 48 h or 1 week. **d** CXCL8 secretion from cells infected with *P. acnes* type II for 48 h or 1 week

The time-dependent differences in secretion of IL-6 and CXCL8 after a *P. acnes* infection were also investigated. PNT1A cells infected with *P. acnes* type IA or II for 48 h had a higher secretion of IL-6 compared to cells infected for 1 week (Fig. 3a-b), although the opposite was seen for CXCL8, where cells infected for 48 h had a lower secretion compared to cells infected for 1 week (Fig. 3c-d). These differences were not statistically significant, although a marginally significant trend could be seen for the CXCL8 secretion from cells infected with *P. acnes* type IA (*p* = 0.055, after adjustments for multiple testing).

## Discussion

In the present study we investigated the presence of *P. acnes* in a case–control study utilizing prostatic tissue obtained from 100 men with PCa and 50 men with no histological signs of the disease. This study design makes the present study the first to our knowledge to simultaneously investigate the prevalence of *P. acnes* in the prostate glands in men with and without histological evidence of PCa. The results indicated that *P. acnes* was more common in men with prostate carcinoma than in healthy men, with *P. acnes* cultured in 60 % of the cases vs. 26 % of the controls (*p* = 0.001). After controlling for age, calendar time and smoking status, presence of *P. acnes* in the prostate was associated with a 4.7 times greater odds of a prostate cancer diagnosis. It was furthermore interesting that among controls found to have tumors, we isolated *P. acnes* from 35 %, an intermediate proportion, providing evidence for *P. acne*s contributing not only to PCa detection but to risk of the disease *per se*. Moreover, *P. acnes* was not related to PSA level, tumor stage, or tumor grade. If *P. acnes* was only facilitating detection, we would expect it to be a marker of more indolent tumors diagnosed at an earlier stage. The prevalence of *P. acnes* in the cystoprostatectomy specimens with prostate cancer was statistically significantly lower than in radical prostatectomy specimens (*p* = 0.01). This result is consistent with *P. acnes* being introduced into the prostate during trans-rectal biopsy in some of the cases, but nonetheless being more frequent in men with undiagnosed PCa than without detectable cancer.

In the present study, we made an effort to minimize the risk of *P. acnes* contamination during tissue sampling and processing in a laboratory setting. Sterile, single-use biopsy needles were used and the tissue cores were obtained in the operating theatre immediately after removal of the prostate. Given that all the samples were handled in the same manner we believe that if our results were influenced by exogenous *P. acnes* it would have similar influence on both cases and controls. Furthermore, the presence of *P. acnes* in the prostate cannot simply be explained by contamination due to biopsies taken at diagnosis, since no trans-rectal biopsies were performed within the controls or controls found to have undiagnosed PCa.

In PCa, both culture and molecular techniques have been used to evaluate the presence of *P. acnes*. In 2005, Cohen *et al*. reported *P. acnes* as the most common microorganism (35 %) in prostate tissue obtained from men with PCa [14]. Alexeyev *et al*., reported that 50 % of radical prostatectomy specimens were positive for *P. acnes* when using fluorescence in situ hybridization [13]. In addition, Sfanos *et al*. performed a comprehensive study evaluating prostatic *P. acnes* infection by applying bacterial culture, 16 s rDNA cloning and, organism-specific PCR. Here, *P. acnes* was the most commonly cultured species (17 %) [16]. The disparity between the studies may be related to use of unequal tissue collection and bacterial detection methods.

Because strain-specific properties may contribute differently to pathogenesis, we genotyped all the bacteria isolates verified as *P. acnes*. In line with previous studies, *P. acnes* type II was the most common type in prostatic tissue in our study, both in cases (51 %) and controls (58 %), when using either repetitive-sequence-based PCR or *tly* sequencing for discrimination. However, the genotyping analyses also revealed the same proportion of *P. acnes* classified as type IA or IB, 42 %, in both cases and controls. These findings suggest that both *P. acnes* types I and II have the capability to colonize the prostate and potentially give rise to a chronic inflammation, a potential risk factor for PCa.

Secretion of cytokines and chemokines, such as IL-6 and IL-8, are of significance for a sustained inflammatory response; these mediators have also been suggested to play an important role in the development of different types of cancers, among them PCa. Therefore, we performed cell-based experiments to investigate if an infection with *P. acnes* affects the secretion of IL-6 and IL-8 from premalignant prostate cell. The cell line PNT1A was infected with two of the *P. acnes* isolates collected from men with PCa in the present study, one type IA and one type II. The results showed that both *P. acnes* types have the capacity to modulate the secretion of IL-6 and IL-8, already at relatively low MOI. These data are supported by previous work performed by Drott *et al*. and Fassi Fehri *et al*., who observed an increased secretion of both IL-6 and IL-8 from the premalignant prostate cell line RWPE1 when infected with *P. acnes* type I [15, 18]. In the present study we have only focused on the secretion of two inflammatory mediators but previous reports have shown that *P. acnes* is an inducer of inflammatory factors such as caspase-1 and reactive oxygen species, giving further support to the suggestion that a sustained *P. acnes* infection may contribute to inflammation related PCa initiation. In line with previous studies we also found a dose response effect between a *P. acnes* infection at different MOI and IL-6/IL-8 secretion. Based on these results, one could speculate that even though a greater number of the bacterium would considerably increase the secreted levels of IL-6 and IL-8, an infection with a smaller number of *P. acnes* may over time be enough to contribute to a micro milieu rich in tumor supporting mediators. These findings can be of importance since *P. acnes* predominantly was cultivated from only two out of six biopsies from each patient.

*P. acnes* may contribute to prostate tumor initiation by mechanisms other than stimulation of inflammatory mediators. In a recent *in vitro* study experiments revealed that exposure to *P. acnes* altered cell proliferation and enabled anchorage-independent growth in infected prostate cells [15]. In the present study we performed wound healing assays to determine differences in cell motility after infection with *P. acnes* type IA and type II. PNT1A cells infected with either type closed the wound faster compared to untreated cells. This effect could not be seen in cells pretreated with Mitomycin C, indicating that *P. acnes*-induced proliferation was the responsible cause rather than increased migration. We believe that even though no statistically significant differences in proliferation was found between infected and uninfected cells in this study, a long-lasting small increase could potentially have an biological impact on prostate cells.

A major strength in the present study was the inclusion of prostatic tissue from men without any histological signs of PCa, addressing a significant limitation in previous investigations. Approximately 40 % of the initial controls showed evidence of PCa, highlighting the challenge of identifying ideal control material with total absence of prostate tumor. Our study also benefitted from a reverse translational approach in which we followed up on the initial epidemiologic findings with basic science approaches to offer more insight into biologic plausibility and underlying mechanisms. Our case–control design did not, however, permit us to ascertain temporality of *P. acnes* infection with respect to PCa. Future studies should also address whether PCa initiation could depend on the expression of different virulence genes in *P. acnes* types present in the prostate gland. Recently, Kasimatis *et al*. reported that a *P. acnes* strain, associated with acne vulgaris, harbors a plasmid. This is the first described *P. acnes* plasmid and this finding could be of importance since the plasmid contained several virulence-associated genes [30].

## Conclusions

The present study provides further evidence for a role of *P. acnes* in prostate tumor development by revealing that men with prostate tumors are more likely to harbor prostatic *P. acnes* compared to men without the disease. Cell-based experiments further indicated that *P. acnes* may be a contributing agent by triggering cell proliferation and IL-6 and IL-8 secretion. If confirmed by prospective studies, these findings could have a profound impact on prevention and future therapeutic strategies.

## Methods

### Prostate tissue sampling

Based on estimates of required sample size for identifying an odds ratio (OR) of 1.75 with 80 % power and 25 % exposure prevalence in controls, we included 100 cases and 50 controls in this study. Cases were men diagnosed with PCa undergoing consecutive radical prostatectomy procedures and controls were men diagnosed with bladder cancer undergoing consecutive cystoprostatectomy procedures. During the latter operation the prostate is removed even though no pathological condition is detected. Because cystoprostectomies are conducted less frequently, controls required a longer timeframe for recruitment. All surgical procedures were conducted between January 2009 and March 2015. The mean age of participants was 64 years (range 53 to 71 years) and 65.7 years (range 42 to 81 years) for cases and controls, respectively. A pathologist examined prostate samples according to the same routine procedure as after a radical prostatectomy and assessed the tissue for PCa without any histological findings. Controls with histological findings of prostate tumor were put as a separate group, controls with PCa. The study was approved by the Ethical Review Boards in Uppsala-Örebro, Sweden (2008/293). All patients were informed about the study before giving written consent.

A biopsy gun and sterile, single-use Biopsy needles (18 ga × 20 cm, ProMagTM Biopsy Needle, Medical Device Technologies, Gainesville, USA) were used to obtain multiple cores from both the right and the left lobes of each prostate. Six biopsies were taken from each patient’s prostate gland according to the schematic illustration in Fig. 1. All biopsies were sampled at the operating theatre immediately after removal of the prostate to minimize the risk of bacterial contamination. The biopsies were placed in tubes containing culture medium and were immediately transported to the Department of Laboratory Medicine, Clinical Microbiology, at the Örebro University Hospital for general cultivation and further characterization of *P. acnes*. The laboratory staffs were blinded to the PCa status of all patients samples.

### Culture diagnostics

The culture and species verification of *P. acnes* was performed in accordance with routine diagnostic procedures. Briefly, the samples were cultured for seven days in an anaerobic atmosphere (80 % N_2_, 10 % CO_2_, 10 % H_2_) at 37 °C on FAA plates (4.6 % LAB 90 Fastidious Anaerobe Agar, LAB M, Lancashire, United Kingdom) supplemented with 5 % horse blood. The isolates were characterized by colony morphology, Gram-staining, catalase and indole tests. All suspected *P. acnes* isolates were further confirmed to species level by API 20 A (bioMérieux, Marcy l’Etoile, France). All isolates were stored at −70 °C in preservation medium (yeast extract; Difco Laboratories, Sparks, USA; and horse serum added to trypticase soy broth [TSB]; BBL, Sparks, MD, USA) pending further analysis.

### Repetitive-sequence-based PCR - DiversiLab

The DNA from the isolates was extracted with the UltraClean Microbial DNA Isolation kit (bioMérieux) following the manufacturer’s instructions. ND-1000 spectrophotometer (NanoDrop Technologies Inc, Wilmington, USA) was used for DNA quantification and all the samples were adjusted to contain approximately 25 to 50 ng/μl. All the samples were amplified using the DiversiLab Propionibacterium Fingerprinting kit (bioMérieux) following the manufacturer’s instructions. Each reaction mixture (25 μl) contained: 18 μl kit-supplied rep-PCR master mix, 0.5 μl AmpliTaq polymerase (Applied Biosystems), 2 μl kit-specific primer mix, 2.5 μl of GeneAmp 10× PCR Buffer, and 2 μl genomic DNA. The PCRs were performed on a thermal cycler (GeneAmpPCR System 9700, Applied Biosystem). The thermal cycling conditions included an initial pre-incubation at 94 °C for 2 min, and 35 cycles of 94 °C for 30 s, 60 °C for 30 s, 70 °C for 90 s, and final extension of 70 °C for 3 min.

To detect the genomic fingerprints of each *P. acnes* isolate, the automated microbial genotyping system (DiversiLab System) was used. The amplified fragments were separated by electrophoresis performed in microfluidics DNA LabChip and detected with an Agilent 2100 Bioanalyzer. One CCUG isolate (CCUG 35749) served as positive control in each round of analysis. The similarity between the isolates was analyzed by the DiversiLab software, version 3.3.40. In this version, the Kullback-Leiber method weighs the presence and absence of peaks rather than peak intensities. The Unweighted Pair Group Method with Arithmetic mean (UPGMA) was used as a clustering method and to create dendrograms and scatter plots [31]. The relatedness was determined by cluster analysis according to guidelines provided by manufacturer. The clustering was defined as a minimum of 95 % similarity, with a difference of up to one band in the dendrogram.

### PCR and sequencing of *P. acnes tly* gene

The DNA from the isolates was extracted with the UltraClean Microbial DNA Isolation kit (bioMérieux) following the manufacturer’s instructions. ND-1000 spectrophotometer (NanoDrop Technologies Inc, Wilmington, USA) was used for DNA quantification and all the samples were adjusted to contain approximately 10 ng/μl. The entire *P. acnes tly* gene was amplified and sequenced by using the previously described *tly* primers PAT-1 (5′-CAGGACGTGATGGCAATGCGA-3′) and PAT-2 (5′TCGTTCACAAGACCACAGTAGC-3′) [25], to generate a 909 bp amplicon. The PCR was performed in a real-time LightCycler system (Roche Diagnostics, Mannheim, Germany) using SYBR Green I fluorescence melting curve analysis for detection of specific amplicon. Each reaction mixture (20 μl) contained: 2 μl Light Cycler FastStart DNA Master SYBR Green I (Roche Diagnostics), 3 mM MgCl_2_, 0.5 μM primers and 2 μl DNA template. The PCR program started with a pre-incubation at 95 °C for 10 min, followed by 40 cycles of 95 °C for 10 s, annealing at 58 °C for 10 s, and 72 °C for 37 s.

Prior to sequencing the PCR products were purified using MultiScreen PCR_μ96_ plate (Millipore, Molsheim, France), according to the manufacturer’s instructions. One microliter of the purified PCR products were then cycle sequenced using 2 μl Big Dye Terminator v3.1, according to the manufacturer’s instructions (Applied Biosystems, Bleiswijk, Netherlands). The cycle sequencing PCR consisted of 25 cycles of 96 °C for 10 s, 58 °C for 5 s, and 60 °C for 4 min. The nucleotide sequences were determined by capillary electrophoreses using an ABI PRISM 3130XL genetic analyzer (Applied Biosystems). The *P. acnes tly* sequences were aligned and compared to GenBank sequences with accession number AY527219 (Type IA), AY644408 (Type IB), AY644409 (Type II), and the *tly* sequence from the reference strain CCUG 35749 (Type III).

### Cell culture

The premalignant prostate epithelial cell line PNT1A was used. PNT1A cells were cultured in RPMI 1640 supplemented with 10 % foetal bovine serum (FBS) and 2 mM L-glutamine and grown in a 37 °C humid atmosphere containing 5 % carbon dioxide.

### Short-term infections

Cells were seeded into T75-bottles with a density of 2 million cells/bottle. The cells were allowed to attach to the flask for 24 h before they were infected with 5, 10, 20, or 50 multiplicity of infection (MOI) of *P. acnes* type IA or II. The two *P. acnes* isolates used were chosen from the *P. acnes* collection cultured and genotyped in this study. Uninfected cells were used as controls. Forty-eight hours post-infection, the cells were harvested and the cell media was frozen at −80 °C until further analyses were performed. The same two *P. acnes* isolates were used in all the cell based experiments.

### Long-term infections

Cells were seeded into T75-bottles with a density of 2 million cells/bottle. The cells were allowed to attach to the flask for 24 h before they were infected with 10 MOI of *P. acnes* type IA or II. Uninfected cells were used as controls. Cells were sub-cultured when they reached 80 % confluence and subsequently re-seeded at a density of 2 million cells/bottle and re-infected with 10 MOI of *P. acnes*. One week post the initial infection, cells were harvested and the cell media was frozen at −80 °C until further analyses were performed.

### Wound-healing and Proliferation assays

For the wound-healing assays, cells were seeded into culture inserts (Ibidi, Munchen, Germany) with a density of 30,000 cells/well and were allowed to attach for 24 h before 4 μg/ml of Mitomycin C were added to each well. One hour after the addition of Mitomycin C, the cells were infected with 5, 10, 20, or 50 MOI of *P. acnes* type IA or II. Uninfected cells as well as cells not treated with Mitomycin C were used as controls. Forty-eight hours post-infection, the culture inserts were removed and the cells were allowed to grow for an additional 48 h, before the closure of the wound was evaluated.

For the proliferation assays, cells were seeded into 96-well plates at a density of 5000 PNT1A cells/well. The cells were allowed to attach for 24 h before infected with 5, 10, 20, or 50 MOI of *P. acnes* type IA or II, uninfected cells were used as controls. Twenty-four and 48 h post-infection, cell proliferation was measured using the CellTiter 96® AQueous One Solution Assay from Promega. The proliferation assays were performed in both technical and biological triplicates.

### Measurement of IL-6 and IL-8 secretion

In order to measure the capability of *P. acnes* type IA and II to modulate the secretion of inflammatory mediators from prostate cells, excreted IL-6 and IL-8 were measured by ELISA. The cell media from the short- and long-term infections was thawed on ice and the samples were analyzed using IL-6 and IL-8 kits (ELISA MAX Deluxe Sets, BioLegend, San Diego, USA) according to the manufacturer’s instructions. The concentration was determined by measuring the optical density at 450 nm on a spectrophotometer (Multiskan Ascent, Thermo labsystems, Helsinki, Finland). All measurements were performed in three technical replicates.

### Statistical analysis

*P*-values for the univariate association between the presence of *P. acnes* and prostate cancer and zonal location of *P. acnes* by case–control status were estimated with Chi-square tests. The independent samples median test was used to determine if the median number of biopsies with *P. acnes* present varied among cases, controls and controls with PCa. Shapiro-Wilk tests were used to investigate if the data from *in vitro* experiments was normally distributed. Logistic regression was used to estimate ORs and 95 % confidence intervals for the association between *P. acnes* and PCa adjusting for age and smoking status.

The non-parametric Kruskal-Wallis test was used to determine if there were differences between groups according to proliferation and secretion. We adjusted for multiple comparisons with a Bonferroni test, where *p* < 0.05 were considered as statistically significant. All *p*-values reported from the Kruskal-Wallis tests are after adjustments for multiple testing. The statistical analyses were performed in IBM SPSS Statistics version 22 (IBM Corp. Armonk, NY, USA).

## Additional file

Additional file 1: Typing results based on *tly* and DiversiLab of *P. acnes* isolates obtained from men with prostate cancer and from men without the disease. (DOCX 28 kb)
