# Erratum to: Frequency and typing of *Propionibacterium* acnes in prostate tissue obtained from men with and without prostate cancer

**DOI:** 10.1186/s13027-016-0084-7

**Published:** 2016-06-21

**Authors:** Sabina Davidsson, Paula Mölling, Jennifer R. Rider, Magnus Unemo, Mats G. Karlsson, Jessica Carlsson, Swen-Olof Andersson, Fredrik Elgh, Bo Söderquist, Ove Andrén

**Affiliations:** Department of Urology, Faculty of Medicine and Health, Örebro University, Örebro, Sweden; Department of Laboratory Medicine, Clinical Microbiology, Faculty of Medicine and Health, Örebro University, Örebro, Sweden; Department of Epidemiology, Harvard T.H. Chan School of Public Health, Boston, MA USA; Channing Division of Network Medicine, Department of Medicine, Brigham and Women’s Hospital and Harvard Medical School, Boston, MA USA; Department of Laboratory Medicine, Pathology, Örebro University Hospital, Örebro, Sweden; Department of Clinical Microbiology, Umeå University, Umeå, Sweden; Faculty of Medicine and Health, Örebro University, Örebro, Sweden; A Member of the Transdisciplinary Prostate Cancer Partnership (TopCaP), Örebro, Sweden; Department of Urology, Örebro University Hospital, SE-701 85 Örebro, Sweden

## Erratum

After publication of the original article [1], it was noticed that Bo Söderquist’s name has been incorrectly spelled as Bo Söderquis. The correct presentation of the author’s name is included in the author list in this erratum and has been updated in the original article [1].
