# Supplementary material for: Frequency and typing of Propionibacterium acnes in prostate tissue obtained from men with and without prostate cancer
Source: Infect Agent Cancer. 2016 Jun 9;11:26. doi: 10.1186/s13027-016-0074-9 (PMC4899914; doi:10.1186/s13027-016-0074-9)
Supplement: Additional file 1: — Typing results based on tly and DiversiLab of P. acnes isolates obtained from men with prostate cancer and from men without the disease. (DOCX 28 kb) [file 13027_2016_74_MOESM1_ESM.docx]

**Additional file 1.** Typing results based on *tly* and DiversiLab of *P. acnes* isolates obtained from men with prostate cancer and from men without the disease.

|  |  |  |  |  |
| --- | --- | --- | --- | --- |
| Pat no. | Pat group | *tly* | Fpp | B no |
| 6 | 1 | II | 9 | 3 |
| 7 | 1 | IB | 2 | 2 |
| 7 | 1 | IA | 2 | 4 |
| 8 | 1 | IB | 1 | 3 |
| 8 | 1 | IB | 2 | 2 |
| 8 | 1 | II | 9 | 1 |
| 8 | 1 | II | 11 | 4 |
| 8 | 1 | II | 11 | 5 |
| 9 | 1 | IA | 2 | 4 |
| 9 | 1 | II | 9 | 5 |
| 12 | 1 | II | 6 | 1 |
| 16 | 2 | II | 2 | 4 |
| 17 | 1 | IA | 2 | 2 |
| 18 | 3 | IB | 2 | 4 |
| 18 | 3 | IB | 3 | 6 |
| 19 | 3 | II | 19 | 3 |
| 21 | 1 | IA | 2 | 1 |
| 22 | 1 | II | 11 | 5 |
| 23 | 2 | IB | 3 | 4 |
| 27 | 1 | IA | 4 | 3 |
| 27 | 1 | IA | 4 | 2 |
| 27 | 1 | II | 21 | 4 |
| 28 | 1 | II | 6 | 4 |
| 29 | 1 | IA | 2 | 5 |
| 30 | 3 | IA | 4 | 4 |
| 32 | 1 | II | 10 | 4 |
| 34 | 1 | II | 6 | 4 |
| 34 | 1 | II | 6 | 2 |
| 34 | 1 | II | 11 | 1 |
| 34 | 1 | II | 11 | 6 |
| 34 | 1 | II | 11 | 5 |
| 35 | 1 | IA | 2 | 4 |
| 35 | 1 | II | 9 | 5 |
| 35 | 1 | II | 11 | 2 |
| 35 | 1 | II | 11 | 6 |
| 36 | 2 | IB | 2 | 1 |
| 40 | 2 | IA | 2 | 5 |
| 42 | 1 | II | 12 | 3 |
| 44 | 1 | II | 7 | 5 |
| 44 | 1 | II | 9 | 6 |
| 44 | 1 | II | 9 | 1 |
| 44 | 1 | II | 11 | 3 |
| 45 | 1 | II | 10 | 2 |
| 48 | 1 | IB | 1 | 6 |
| 48 | 1 | III | 23 | 1 |
| 48 | 1 | III | 23 | 3 |
| 52 | 1 | IA | 2 | 5 |
| 52 | 1 | IA | 2 | 5 |
| 52 | 1 | IA | 2 | 4 |
| 52 | 1 | IA | 2 | 6 |
| 53 | 1 | IA | 2 | 5 |
| 53 | 1 | IA | 2 | 4 |
| 53 | 1 | II | 6 | 5 |
| 53 | 1 | IA | 13 | 1 |
| 54 | 1 | IA | 2 | 1 |
| 55 | 1 | IB | 1 | 6 |
| 55 | 1 | II | 16 | 5 |
| 55 | 1 | II | 15 | 1 |
| 56 | 1 | IB | 2 | 3 |
| 57 | 1 | II | 8 | 6 |
| 58 | 1 | II | 6 | 1 |
| 58 | 1 | II | 6 | 6 |
| 62 | 1 | IB | 2 | 1 |
| 62 | 1 | IA | 2 | 3 |
| 62 | 1 | II | 6 | 4 |
| 63 | 1 | II | 6 | 2 |
| 63 | 1 | II | 6 | 1 |
| 69 | 1 | IA | 2 | 5 |
| 71 | 1 | II | 9 | 2 |
| 71 | 1 | II | 9 | 3 |
| 73 | 2 | II | 6 | 1 |
| 73 | 2 | II | 6 | 4 |
| 75 | 1 | II | 9 | 5 |
| 75 | 1 | II | 9 | 4 |
| 78 | 2 | IA | 2 | 2 |
| 79 | 3 | IA | 13 | 3 |
| 80 | 2 | II | 12 | 4 |
| 80 | 2 | II | 12 | 2 |
| 80 | 2 | II | 12 | 1 |
| 80 | 2 | II | 7 | 5 |
| 80 | 2 | II | 12 | 3 |
| 80 | 2 | II | 15 | 6 |
| 81 | 1 | IA | 2 | 4 |
| 84 | 1 | III | 22 | 3 |
| 84 | 1 | III | 22 | 2 |
| 86 | 1 | IA | 2 | 4 |
| 86 | 1 | II | 11 | 5 |
| 87 | 3 | IB | 2 | 5 |
| 89 | 1 | III | 23 | 6 |
| 93 | 3 | IA | 2 | 3 |
| 93 | 3 | IA | 2 | 2 |
| 93 | 3 | IA | 2 | 5 |
| 93 | 3 | IA | 2 | 4 |
| 93 | 3 | IA | 2 | 1 |
| 93 | 3 | IA | 2 | 6 |
| 94 | 1 | IB | 5 | 4 |
| 96 | 1 | II | 6 | 2 |
| 96 | 1 | II | 6 | 6 |
| 96 | 1 | II | 6 | 5 |
| 96 | 1 | II | 6 | 4 |
| 96 | 1 | II | 6 | 1 |
| 96 | 1 | III | 22 | 3 |
| 97 | 1 | IA | 2 | 2 |
| 97 | 1 | IB | 2 | 1 |
| 97 | 1 | II | 10 | 3 |
| 98 | 1 | II | 10 | 1 |
| 99 | 1 | II | 10 | 1 |
| 99 | 1 | II | 17 | 3 |
| 99 | 1 | II | 18 | 6 |
| 100 | 1 | IA | 2 | 5 |
| 100 | 1 | II | 15 | 1 |
| 100 | 1 | II | 12 | 6 |
| 100 | 1 | II | 17 | 4 |
| 100 | 1 | II | 20 | 3 |
| 104 | 3 | IA | 5 | 6 |
| 104 | 3 | II | 12 | 3 |
| 104 | 3 | IA | 13 | 2 |
| 108 | 1 | IB | 1 | 4 |
| 108 | 1 | IB | 1 | 1 |
| 108 | 1 | IB | 1 | 3 |
| 108 | 1 | II | 6 | 2 |
| 109 | 1 | II | 6 | 5 |
| 109 | 1 | II | 10 | 2 |
| 113 | 1 | III | 22 | 1 |
| 115 | 2 | IA | 2 | 2 |
| 116 | 1 | IB | 1 | 4 |
| 116 | 1 | IB | 5 | 1 |
| 116 | 1 | II | 9 | 3 |
| 116 | 1 | II | 9 | 2 |
| 116 | 1 | II | 16 | 6 |
| 120 | 1 | II | 6 | 2 |
| 120 | 1 | II | 12 | 1 |
| 120 | 1 | II | 21 | 5 |
| 121 | 1 | II | 6 | 5 |
| 122 | 1 | IA | 2 | 5 |
| 122 | 1 | II | 11 | 2 |
| 122 | 1 | III | 23 | 4 |
| 123 | 1 | IA | 2 | 1 |
| 124 | 1 | II | 9 | 2 |
| 126 | 1 | IB | 1 | 2 |
| 126 | 1 | IB | 1 | 1 |
| 126 | 1 | IB | 1 | 5 |
| 126 | 1 | IB | 1 | 6 |
| 126 | 1 | IB | 1 | 3 |
| 127 | 1 | IA | 2 | 3 |
| 127 | 1 | IA | 2 | 4 |
| 128 | 1 | IA | 2 | 6 |
| 128 | 1 | IA | 2 | 1 |
| 128 | 1 | IA | 2 | 2 |
| 131 | 1 | IA | 2 | 5 |
| 132 | 1 | IA | 2 | 1 |
| 132 | 1 | IA | 2 | 4 |
| 135 | 1 | IA | 2 | 2 |
| 136 | 1 | IA | 2 | 4 |
| 147 | 1 | IA | 2 | 1 |
| 147 | 1 | II | 9 | 6 |
| 148 | 1 | III | 23 | 5 |
| 149 | 1 | IB | 1 | 1 |
| 149 | 1 | II | 9 | 3 |
| 149 | 1 | II | 9 | 4 |
| 149 | 1 | III | 12 | 5 |
| 149 | 1 | III | 22 | 6 |
| 150 | 1 | IA | 2 | 3 |
| 152 | 3 | IA | 2 | 2 |
| 156 | 2 | II | 15 | 5 |
| 160 | 2 | II | 6 | 3 |
| 163 | 2 | IA | 2 | 2 |
| 171 | 2 | IB | 3 | 3 |
| 174 | 3 | IB | 3 | 1 |
| 174 | 3 | IB | 3 | 4 |
| 174 | 3 | IB | 3 | 5 |
| 176 | 2 | IB | 3 | 1 |
| 177 | 3 | IB | 3 | 3 |
| 177 | 3 | IB | 3 | 6 |
| 184 | 3 | IB | 3 | 4 |
| 184 | 3 | IB | 3 | 5 |
| 184 | 3 | IB | 3 | 6 |
| 185 | 3 | II | 14 | 2 |
| 185 | 3 | II | 14 | 3 |
| 185 | 3 | II | 14 | 4 |
| 185 | 3 | II | 14 | 5 |
| 189 | 3 | II | 9 | 1 |
|  |  |  |  |  |

*Pat= Patient, Group; 1= Case, 2= Control, 3= Control with prostate cancer, FPP= Fingerprint pattern, B no= Locations of the biopsies (see Figure 1).
